# A qualitative enquiry into dental students’ perceptions of dentistry as a career choice in the State of Qatar

**DOI:** 10.1186/s12909-022-03522-4

**Published:** 2022-06-11

**Authors:** Alaa Daud, Manal Matoug-Elwerfelli, Xiangyun Du, Kamran Ali

**Affiliations:** 1 College of Dental Medicine, QU Health, Qatar University, Doha, Qatar; 2 Aalborg UNESCO Center for Problem-Based Learning, Department of Planning, Aalborg University, Rendsburggade 14, 9000 Aalborg, Denmark

**Keywords:** Dental profession, career motives, dental college admissions, patient care, hands-on skills, dental education

## Abstract

**Background:**

Career choice is a complex, multifaceted process affecting all aspects of life. Motivational factors of aspiring dentists are crucial to inform institutions and aid with the admission process. The aim of this study was to explore undergraduate dental students’ perceived motivation for their career choice in the first dental institution in the State of Qatar to bridge the gap in knowledge in this area.

**Methods:**

Homogeneous purposive sampling technique was employed to collect data from all year 2 and year 3 undergraduate dental students to gain a deeper insight into their motivation of career choice. An explorative qualitative method using face-to-face focus group sessions were utilized. All focus groups were conducted in English and contained a moderator and observer. A topic guide was used to ensure data collection standardization. Participants’ views were recorded and filed notes obtained. Data was transcribed and analysis performed utilizing an inductive thematic approach.

**Results:**

A total of 34 students (89.5%) from year 2 and 3 participated in the focus groups. Data analysis revealed six main themes emerging from this study namely; altruism and patient care, family influence factors, childhood aspirations, hands-on practical skills, professional and social status, and the opportunity to reconsider and transfer. Based on frequency, altruism, opportunities to learn hands-on practical skills and professional status appeared to be the main drivers influencing students’ choice of dentistry as a professional career.

**Conclusion:**

This qualitative study presents the first national study providing insightful information regarding current undergraduate dental students’ decision process in relation to their profession selection, and shows that opportunities to provide patient care seems to be the key to motivation. There was also a strong inclination towards performing hands-on practical tasks as a dentist, and developing a professional status. Interestingly, financial reward did not feature as a motivational factor in this study. The study highlights the influence of socio-cultural and economic factors on choosing dentistry as a career. This data could help dental institutions better understand future applicant’s motivations to join dentistry and assist with the academic recruitment/admission process and targeting students with a potential to excel in the profession.

## Introduction

Career choice is a complex, multifaceted process affecting all aspects of life. Selecting a career option is one of the most important decisions an individual makes. Students entering higher education may end up choosing a particular field of study regardless of their interests and talents [1–3]. A wrong choice, however, may lead to lack of student motivation in that field of study, inducing mental health issues. Thereafter, struggling to succeed in that field may result in a waste of human and economic resources.

Motivation provides an individual with power and direction to progress. Educational motivation is a prerequisite to learning and is a factor that helps the learner maintain continuity and enjoy the learning experience [4]. The performance of health systems have recently been intimately linked with the motivational skills and commitment of health care professions [2]. Research into the motivation and decision making of the emerging health workforce is crucial to inform government and policy makers. This will enable a better understanding of recruiters and support systems to nourish the health-care system with a well-motivated workforce.

Over the decades, more attention has been paid to the key role of dentists in improving the quality of dental care through preventing, diagnosing and treating oral disease, and further extends to educating patients and changing behavior. Despite the expansion of services and the technological improvements, oral health inequalities continue to persist and the demand for qualified dentists remain a national target [5]. Since the beginning of the 21^st^ century, the supply of dentists per capita has expanded and is projected to increase further [6]. Many factors have been associated with the attraction of high school graduates to the dental profession. These could be divided into external factors, indirectly affecting human choice, such as family influence, educational environment, social-media and socio-economic factors, and personal factors, controlled by the individual, such as status, interests, security and care for patients [2, 3, 6, 7].

The dental profession is currently facing a time of unprecedented change, with continual advancement in techniques, materials and products [8], a significant shift of dental care to the private sector [9], an increased emphasis on skill mix development within the dental team [10], and a significant level of study debt [6, 11]. Moreover, there is some evidence of generational effects in the workplace, suggesting different expectations of the workforce compared to those from previous generations [12]. Nonetheless, graduate applications for entry into dental programs remain heavily oversubscribed [2].

Overtime, authors have explored a wide range of motivational factors underlying applicants’ career choices. Most of the published research however, has been survey-based, using different instruments, and pre-set “author” designed questions, generating findings that are not necessarily comparable [2]. Qualitative research mainly in the form of interviews and focus groups are increasingly utilized and regarded as common methods of data collection in dentistry [13] and provide a deeper understanding of participants' beliefs and perspectives [14].

Limited data is available concerning career choice in developing countries, specifically within the Gulf Cooperation Countries. The State of Qatar has recently established the first Dental College in 2019. The dental program lasts 6 years and entrance to the program depends on the results of General Secondary Education Certificate or equivalent certificate based on the British or American curriculum. Admission criteria include a high level of academic performance, including internationally recognized tests in English and mathematics. Candidates are ranked competitively, shortlisted and then invited to a structured interview. It has been suggested that admission methods evaluating motivation could help dental schools better understand applicant’s interest in the profession, help select future successful practitioners, retain a motivational workforce and support personal professional development [7, 15]. The investigative process of motivation will also avoid admitting students who may be less interested in this career, and may not fulfil the wide range of requirements of being a competent future clinician [2, 3, 7, 15]. The current undergraduate dental students will be classified as the sole home trained dentists to the profession in Qatar, thus the views of the initial student cohorts might have a significant impact on the profession selection of future cohorts. Therefore, there is a need to investigate this phenomenon and bridge gaps in the knowledge in this area.

The aim of this study was to explore undergraduate dental students’ perceived motivation for their career choice, using qualitative focus group data collection measures to shed further light and generate a deeper and richer understanding. The outcomes of this study will be used to advance knowledge regarding motivations in this region, to help inform stake-holders and facilitate the admission process.

## Methods

### Study design

A qualitative study design using focus groups was deemed appropriate and applied to this study, as the aim was to explore, in-depth, the perceptions of dental students. Ethical approval was obtained by the institutional review board at Qatar University (IRB No. QU-IRB 1596-EA/21). Focus group sessions as data collection methods was utilized to uncover dental students’ perceptions and values for choosing dentistry as a career, and identify emerging patterns in a digital generation, encompassing those who were born into or raised in the digital era, with wide-spread access to modern-age technology. This methodological approach was selected to gain a deeper insight into an undergraduate dental program with a relatively small cohort size [16]. Homogenous purposive sampling technique was undertaken to recruit all dental students, in year 2 (n = 24) and year 3 (n = 14) of an undergraduate program at the newly established College of Dental Medicine, Qatar University, as their perspectives or experiences were relevant to the study question. It was deemed that year 2 and 3, rather than year 1 students, have the potential to provide rich, relevant and diverse data pertinent to the research question. The authors acted as gatekeepers for the research invites.

### Data collection

All undergraduate year 2 and 3 dental students, Qatari and non-Qatari, were invited to participate and received official emails notifying them of the aim of the study including a participant information leaflet. Participation in the study was voluntary, and a prior written consent was obtained. All participants were reassured that responses will be anonymous and dealt with confidentially. The authors carefully planned the face-to-face focus group sessions, the venue for the focus group session was accessible and convenient to all participants and booked beforehand. Spacious rooms within the dental college, with enough light and ventilation, were selected to conduct the sessions. Semicircular or circular seating was designed to allow participants to see, listen to and engage with one another during the discussions [16].

A topic guide was developed for the focus group discussions to standardize data collection and help keep the discussion relevant to the research aim. The guide evolved based on a discussion with the research team and a literature review. It contained open-ended and neutral probing questions to avoid socially desirable responses. Guiding questions included why students selected dentistry as a career profession, what were the motivations/drivers, any internal or external factors directly or indirectly influencing their choice, and their perception regarding dentistry as a career choice? All face-to-face focus groups were conducted in English, contained a moderator and observer, and lasted approximately 60- 90 min each, to avoid participant fatigue, but ensure comprehensive data collection. Issues emerging from group discussions were probed further and participants were encouraged to elaborate on their responses further [17]. The study was conducted during November 2021.

### Data analysis

Focus groups were audio-recorded and transcribed. Data was cleaned and returned to participants for any comments. Six main stages to analyze data were followed in this study; familiarization, coding, finding themes, reviewing themes, synthesizing and defining themes, and producing a final report [18]. The process began by familiarization of the researchers with the raw data in transcription to develop a conceptual framework of themes and sub-themes. The data was analyzed manually and an inductive, thematic data analysis approach was undertaken for this study [19]. This method was adopted to generate rich data on the topic. The inductive approach derives the thematic framework from actual collected data and is considered the most commonly used method in dental qualitative research [20]. The next stage involved further abstraction to define the elements to a higher level and refine categories [15]. AD and MME read the transcripts independently and coded text that was considered relevant to the research, for their manifest and latent content, to ensure trustworthiness of the data analysis [18]. Throughout the analysis, findings were linked back to the original transcripts, ensuring that the emerging findings accurately reflect the context and perceptions expressed. A preliminary and secondary list of codes was drawn up by both researchers and once an agreement was reached, the main codes were produced. During this process, common phrases presenting the same idea or meaning were identified. Other team members (KA and XD) validated the aforementioned generated codes. Once all data were coded, codes were compared and sorted into relevant themes and subthemes. Themes are defined as something that has a certain level of pattern or meaning in relation to the research questions in the data) [18]. Themes extracted were discussed by the research team and any discrepancies were resolved. Quotes representing the themes were finally selected based on agreement of the whole research team. The following results are reported according to the Consolidated Criteria for Reporting Qualitative Studies (COREQ) [17].

## Results

**Fig. 1:**
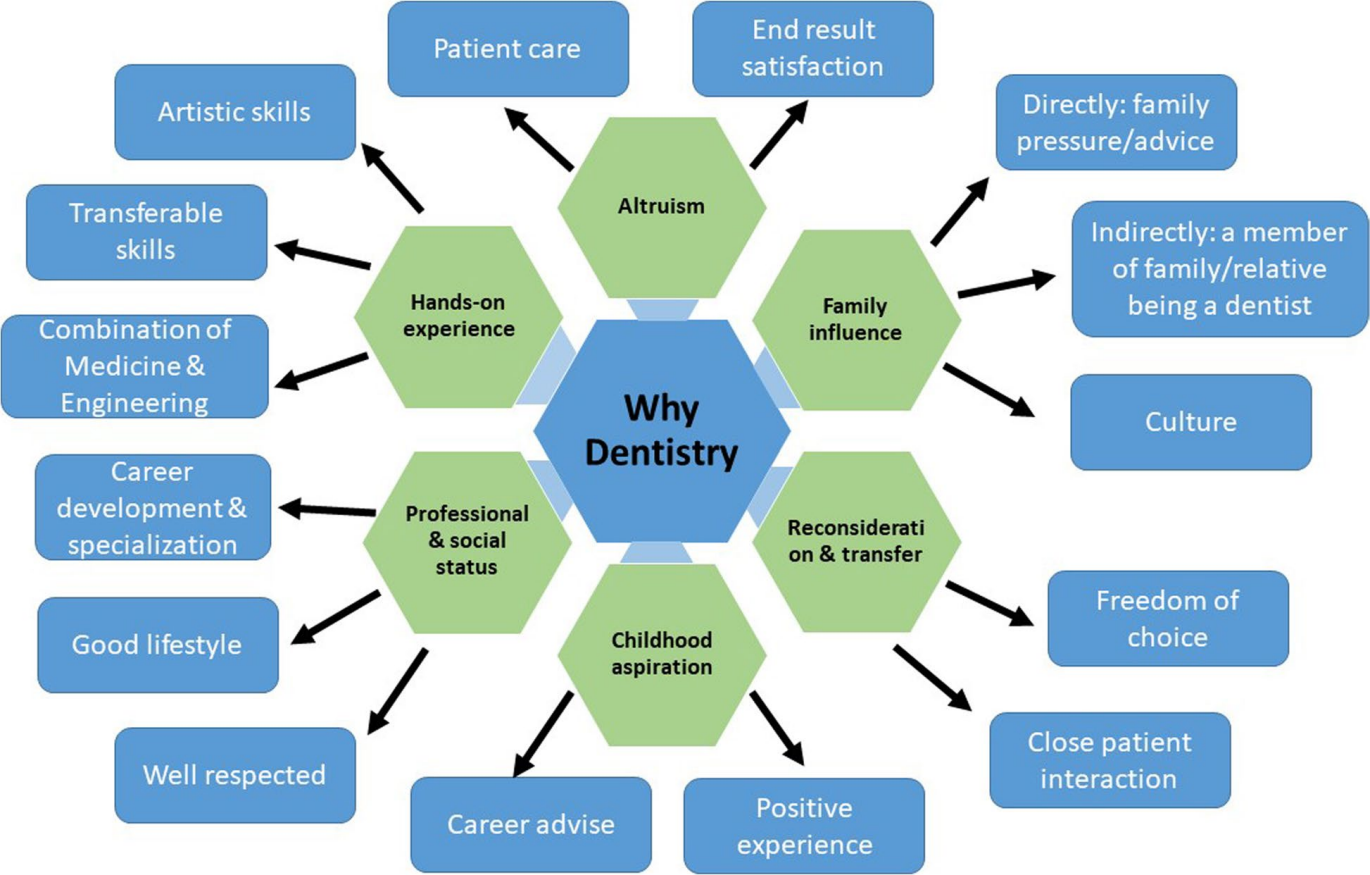
Key Themes and Subthemes for Dentistry as a Career Choice

Demographics of participants included age ranging from 19 to 22 years old, and gender distribution of: Year 3 (n = 14) had 2 groups, one with 7 females, no male participants, and the other with five females and 2 male participants. Year 2 (n = 20) included 3 groups; the first with 7 females and 2 male participants, the second with 3 females and 1 male participant, and the third with 5 females and 2 male participants. Findings from the analysis are depicted in Fig. 1 (Key Themes and Subthemes for Dentistry as a Career Choice). Overall, six major themes were identified with accompanying subthemes. Choices of participating students of dentistry as a professional career were influenced by the following:

### Theme 1. Family influences

A strong family influence guiding students towards their future career direction was evident among the participants. Some students admitted that applying for dentistry was not purely their decision; they were encouraged by their families to consider Dentistry as a career. Delving into that, students felt influenced by their family because either a family member is a dentist, family thought the student had the required skills to become a dentist, or the cultural repute of dentists being highly regarded.

“My father wanted me to study dentistry to be respected” (Student 3, year 2).

“I think what motivated me was my uncle is a dentist, so I was like I want to pursue this career too” (Student 4, year 3).

“My family always telling me that I have good hand skills and should apply for dentistry” (Student 1, year 2).

### Theme 2. Opportunities to develop hands-on practical skills

The choice of a dental career profession related to the art and science of dentistry was evident. Out of the cohort of participating students, 35% (n = 12) showed interest in dentistry for being a hands-on practical career. Some were attracted to the “applied” aspects of dentistry:

“I didn't apply to medicine or any other healthcare profession because when watching YouTube videos on dentistry and how they do things, when carving wax for example, that really intrigued me.” (Student 4, year 2).

“I am good at drawing, and I think dentistry is art and that’s why I applied” (Student 2, year 2).

“I like doing things with my hands, so I chose dentistry” (Student 1, year 2).

Interestingly, the perception of some students revealed that dentistry is a mixture of medicine and engineering, and hence, has the advantage of combining the health knowledge and patient interaction of medicine, with the art and science of engineering.

“I liked engineering and medicine, but when I saw dentistry, it combined both in terms of hands-on practice and patient care” (student 6, year 2).

“When I explored dentistry, I realized it has a promising future and integrates both art and medicine and science technology to take care of an individual person. It also has the aesthetic perspective” (Student 2, year 3).

### Theme 3. Childhood aspirations

The aspiration to dentistry from a young age, paving the path for this chosen discipline was evident among the participants. Students reported examples such as, acquiring a positive experience at the dentist as a child. This experience made them consider dentistry as a career.

“Actually, it was my childhood dream to become a dentist” (Student 7, year 2).

“I have always wanted to be in a healthcare profession from the beginning and dentistry was my option” (Student 8, year 2).

“I had a terrific experience of dentists since childhood” (Student 7, year 3).

### Theme 4. Professional and social status

Dentistry, as a profession, is ranked as a highly respected and well-paid career. Within the study participants, dentistry as a health care profession was perceived to be a prestigious career, highly respected. Interestingly, none of the participants in this study raised the financial income as a motivational reason for selecting this career.

“I prefer dentistry for many little reasons, one of which is the identity” (Student 6, year 2).

“It's a new college in Qatar, and I thought it's a very nice opportunity.. making my country proud” (Student 1, year 2).

“I realized it has a promising future.. people will respect me” (Student 3, year 3).

“For me, being a dentist is prestigious” (Student 9, year 2).

### Theme 5. Altruism factors

The privilege in providing patient care and working with people was also frequently mentioned within the cohort of students. The participants perceived dentistry as a “people oriented” profession, where they would have direct contact with the patient and feel rewarded.

“I transferred from pharmacy as I always wanted to have more communication and contact with the patients and be able to treat them” (Student 7, year 3).

“I want to give people a nice smile” (Student 3, year 3).

“I think I’m a pure extrovert; I enjoy socializing with people, caring for them … and the dental profession is a people-oriented profession” (Student 5, year 3).

### Theme 6. The opportunity to reconsider career choice and transfer

At Qatar University, once students are accepted on a particular “Major” and enroll in the first year, they have the opportunity to apply for a transfer to a different college within the university if they meet the requirements at the end of the first academic year. Therefore, if a student realizes in their first year of a particular college that they have made the wrong choice, they might be able to transfer provided places are available too.

“I found medicine boring” (Student 5, year 2).

“Dentistry is more focused on one thing which is the oral cavity, but medicine covered a lot of topics” (Student 9, year 2).

“Engineering has a lot of physics and math, so I preferred a healthcare profession like dentistry; I wanted something to be really practical” (Student 1, year 3).

“Pharmacy was not as hands-on as dentistry” (Student 2, year 2).

## Discussion

An individual’s career choice is a crucial long-life made decision, most likely to have a direct impact on their future, wellbeing and the role they play in the society. The processes leading to a career choice has been described on the basis of individual differences in aptitudes and personality, in addition to sociostructural conditions, emphasizing social class and opportunity structures [21].

Motivational factors of aspiring dentists have been investigated over the years [1–3, 6, 7, 22–28] and the aim of this study was to explore undergraduate dental students’ perceived motivation for their career choice in Qatar, to shed further light, and generate a deeper a understanding. Although survey-based studies investigating academic dental students’ motivation towards career choice have been previously conducted; this study used qualitative methods to gain a deeper insight into the decision-making processes guiding career choices for dental students. Qualitative research mainly in the form of interviews and focus groups are increasingly utilized and regarded as common methods of data collection within the dental field [13] and provide a deeper understanding of participants' beliefs and perspectives [14].

Overall, results of this study highlighted a range of opinions related to individual motivations to pursue a career in dentistry. One can observe from the gender distribution of the participating cohorts, that the majority of students were females. This is dictated by the rising number of female applicants to study dentistry in Qatar; a phenomena taking place world-wide [15, 29–31]. Gender balance of the dental profession has changed considerably over the last 2 decades. In Europe for example, females accounted for more than 60% of all practicing dentists in 2016, with almost four in five dentists in Finland, Russia, Latvia and Lithuania being female [29, 32]. The high percentage of females in this study is comparable to others [26, 33, 34].

Self-motivation was found to be a key motivation for a career in dentistry in Germany and Finland [7]. In this study, equivalent motives emerged, nevertheless, family influence, from parents or family relatives who are dentists, was also a commonly reported theme. Family impact and desire for their young adults to enter a dental profession has also been previously reported elsewhere [23, 24]. In a survey of two dental school applicants in the United Kingdom (UK), over 45% of the applicants reported relatives who were either dentists or doctors [25]. A previous study has shown that in addition to different motivational factors to opt for dentistry, education levels of parents influenced the students’ motivation in choosing their field of study [3]. This factor was less remarkable amongst the participants in this study.

Acquisition of hands-on practical skills was also highlighted as a reason influencing students’ career choice. Clinically, in order to perform dental procedures, a dentist must be adept to work with precision on an extremely small scale. Additionally, superior eye-hand coordination is crucial to ensuring the safety of patients and the integrity of the profession [35]. This study exposed a strong link, as perceived by students, between art, manual dexterity and the profession of dentistry. These findings corroborate with results from a previous study that found a significant correlation between the different tests, written questions and the drawing test. Moreover, the best students in hands-on practical dental work during the second year of study were found to be the ones who were best at both the drawing test and the ‘medical concepts’ examination, of which the latter was the only test needing reflection [36]. Conversely, Giuliani et al., reported that basic manual dexterity is not essential in the selection of dental students. Students who could follow training significantly improved in their manual ability [37].

Medicine and dentistry are intimately connected, however, at an undergraduate level, medical students are expected to perform basic practical skills, such as performing electrocardiography (ECG), delivering intravenous (IV) and intramuscular injections (IM), suturing superficial wounds and Bladder catheterization [38]. In comparison, dental students require acquisition of fine-hand dexterity at an early stage of their undergraduate studies, in a confined space; namely, the oral cavity [39]. Engineering on the other hand, may require practical skills, depending on the different disciplines (mechanical, chemical, civil, and electrical engineering, to name a few). Nonetheless, they do not require communicating and interacting with patients. Interestingly, students in this study perceived the dental profession to be the best of both worlds, combining both medicine and engineering, hence integrating hands-on practical skills and patient care.

The interest of the currently explored “generation Z” in hands-on practical tasks could also stem back to them being a “digital” generation. Using computers, texting on their phones with others, and playing video games are all activities that have been found helpful in improving manual dexterity [40]. Evidence has established that previous video and smartphone gaming experiences might have a positive impact on hand–eye coordination and visuospatial skills [41, 42]. Therefore, it is important to recognise that the current generation may have very different skill-sets and expectations to those of earlier generations. Research indicates that this variation in expectations has implications for health professions and the performance of health systems [15, 43, 44].

Childhood aspiration and general interest in dentistry was reported within the study cohort as a common theme behind individual’s career choice. Interestingly, previous evidence has reported that occupations are chosen at an early age, especially in the context of a scientist career in comparison to other career directions [21, 45].

Dentistry, as a highly respected profession, with a social status and a prestigious career was also reported within the study cohort as a reason behind an individual’s career selection. In line with previous questioner based studies, a professional job related factor was evident [27, 33, 34]. Statistics predict the overall employment of dentists will grow by 7% from 2018 -2028, faster than average, and the demand for them will increase as the population ages and as research continues to link systemic health to oral health [46]. According to *U.S. News and World Report*’s annual “list of the 100 best jobs”, dentists and orthodontists are frequently named two of the best jobs. [47]. Flexible working hours, financial gain and job security are closely related to professional job related factors [2]. Previous survey based studies in countries such as the UK, India, USA, Iran, South Africa, Australia and Syria, highlighted financial status and security as two of the main reasons behind dental career selection [7, 26, 27, 33]. However, these aspects were not reported by participants in this study. This may be attributed to the fact that the economy of Qatar is one of the richest in the world based on GDP per capita, ranking generally amongst the top 10 richest countries worldwide [48]. Qatar nationals, forming 70% of the current year 2 and year 3 cohort, come from a wealthy background. Hence, it is not surprising that financial considerations do not appear to be a predominant determinant of choosing dentistry as a career for the participants. This is similar to non-Qatari residence with high income. On the contrary, educational costs may deter students from applying to dentistry in other countries [2, 6, 27, 33, 49, 50].

Altruism is a term referring to the practice of selfless concern and devotion to others; in simple words, the desire to help, and act to promote someone else’s welfare [51]. The most common motive relayed by students (55%) in this study was altruism. This is not surprising since dentistry is a healing science [52]. This is similar to the findings of other studies conducted in Sweden, Japan, Jordan, Iran and Australia [23, 34, 53, 54] whereby dental students are motivated to help people and promote better oral health.

The ability to reconsider one’s initial choice and the option to transfer between colleges is an advantage. Within the participating cohort, a transfer specifically from either the medical or pharmacy colleges was recorded. Despite the scientific nature of the above-mentioned colleges, dental career path was favored and reconsidered due to the hands-on practical nature and close patient interaction within the dental profession. On the contrary, previous surveys have reported that dental college was the second favored choice, with medicine in the first rank, among the participating dental students [25]. Although previous studies report flexible and appropriate working hours as an advantage in the dental profession in comparison to medical profession [7], none of the current participating students reported on this.

The primary strength of this study is that all current undergraduate dental students participated in this qualitative research conducted via face-to-face focus group sessions. To further strengthen the methodological aspect, each session was conducted with a presence of two researchers; a moderator and observer. Additionally, no previous study of this nature has been looked into in the State of Qatar. However, this study only included views of a sole dental school and the small number of currently enrolled undergraduate students are recognized limitations, limiting the generalizability of study findings.

### Strengths and limitations

This is the first national study in Qatar exploring dental students’ motivations for their career choice. The findings provide baseline data to inform the admission process.

A range of opinions on the ideal size of focus groups has been reported in the literature ranging from 4 to 12 participants to yield diversity in information provided and allow in-depth exploration [19, 55, 56]. In line with the contemporary guidelines in qualitative research, the size of the focus groups in this research ranged between 4 to 9 participants. Focus groups provided an opportunity for participants to listen to their peers views and experiences for self- reflection. On the other hand, desirability bias amongst students to share their views and experiences in the presence of their peers may have influenced their participation.

The main limitation of the study is that we did not explore the influence of demographic factors, particularly gender, on choosing dentistry as a career. Future research could monitor the participants longitudinally to evaluate any correlations between motivation and academic performance.

## Conclusion

This qualitative study presents the first national study providing insightful information regarding current undergraduate dental students’ decision process in relation to their profession selection, and has concluded that students perceived their motivation to be mainly related to patient care. There was also a strong inclination towards performing hands-on practical tasks as a dentist, and developing a professional status. Interestingly, financial reward did not feature as a motivational factor in this study. This data could help dental institutions better understand future applicant’s motivations to join dentistry and assist with the academic recruitment/admission process and targeting students with a potential to excel in the profession.

## Data Availability

The data that supports the findings of this study are available on request from the corresponding author. The data are not publicly available due to ethical restrictions.

## References

[1] Gilavand A (2016). The comparison of Iranian and Foreign students’ motivations to choose dentistry field of study. Int J Pediatr.

[2] Gallagher JE, Patel R, Donaldson N, Wilson NHF (2007). The emerging dental workforce: Why dentistry?. A quantitative study of final year dental students’ views on their professional career, BMC Oral Health.

[3] A. Gilavand, G. Barekat, M. Hosseinpour, Evaluation of Dental Students’ Motives and Viewpoints on Their Career Choice in Ahvaz, Jentashapir J. Heal. Res. 6 (2015). 10.17795/jjhr-30384.

[4] Rice CD, Hayden WJ, Glaros AG, Thein DJ (1997). Career changers: dentists who choose to leave private practice. J Am Coll Dent.

[5] Lala R, Gibson BJ, Jamieson LM (2021). The Relevance of Power in Dentistry. JDR Clin Transl Res.

[6] Nasseh K, Vujicic M (2017). The relationship between education debt and career choices in professional programs: The case of dentistry. J Am Dent Assoc.

[7] Du Toit J, Jain S, Montalli V, Govender U (2014). Dental Students’ Motivations for Their Career Choice: An International Investigative Report. J Dent Educ.

[8] Duke ES (2003). Has dentistry moved into the nanotechnology era, Compend Contin. Educ Dent.

[9] The Information Centre (2006). Dental earnings and expenses report, 2004/05, London Dep. Heal..

[10] Department of Health (2002). NHS Dentistry: Options for Change, London Dep. Heal.

[11] Davidson PL, Nakazono TT, Carreon DC, Gutierrez JJ, Shahedi S, Andersen RM (2011). Reforming dental workforce education and practice in the USA. Eur J Dent Educ.

[12] Dittman M (2005). Generational differences at work. Monit Psychol.

[13] Gill P, Baillie J (2018). Interviews and focus groups in qualitative research: An update for the digital age. Br Dent J.

[14] Nyumba TO, Wilson K, Derrick CJ, Mukherjee N (2018). The use of focus group discussion methodology: Insights from two decades of application in conservation. Methods Ecol Evol.

[15] Gallagher JE, Clarke W, Eaton KA, Wilson NHF (2007). Dentistry - A professional contained career in healthcare. A qualitative study of Vocational Dental Practitioners’ professional expectations, BMC Oral Health.

[16] M. Gundumogula, Importance of Focus Groups in Qualitative Research, Int. J. Humanit. Soc. Stud. 8 (2020). 10.24940/theijhss/2020/v8/i11/hs2011-082.

[17] Tong A, Sainsbury P, Craig J (2007). Consolidated criteria for reporting qualitative research (COREQ): A 32-item checklist for interviews and focus groups. Int J Qual Heal Care.

[18] Braun V, Clarke V (2006). Using thematic analysis in psychology. Qual Res Psychol.

[19] Chai HH, Gao SS, Chen KJ, Duangthip D, Lo ECM, Chu CH (2021). A concise review on qualitative research in dentistry. Int J Environ Res Public Health.

[20] Ritchie J, Spencer L, O’Connor W (2003). Carrying out qualitative analysis. Qualitative research practice: a guide for social science students and researchers.

[21] Schoon I (2001). Teenage job aspirations and career attainment in adulthood: A 17-year follow-up study of teenagers who aspired to become scientists, health professionals, or engineers. Int J Behav Dev.

[22] Michalec B, Giordano C, Arenson C, Antony R, Rose M (2013). Dissecting first-year students’ perceptions of health profession groups: Potential barriers to interprofessional education. J Allied Health.

[23] Karibe H, Suzuki A, Sekimoto T, Srithavaj MLT, Iamaroon A, Warita S, Kawakami T, Ogata K, Shirase T, Nakahara S (2007). Cross-Cultural Comparison of the Attitudes of Dental Students in Three Countries. J Dent Educ.

[24] Orenuga OO, da Costa OO (2006). Characteristics and Study Motivation of Clinical Dental Students in Nigerian Universities. J Dent Educ.

[25] Stewart FMJ, Drummond JR, Carson L, Hoad Reddick G (2004). The future of the profession - A survey of dental school applicants. Br Dent J.

[26] Hallissey J, Hannigan A, Ray N (2000). Reasons for choosing dentistry as a career - a survey of dental students attending a dental school in Ireland during 1998–99. Eur J Dent Educ.

[27] Crossley ML, Mubarik A (2002). A comparative investigation of dental and medical student’s motivation towards career choice. Br Dent J.

[28] Scarbecz M, Ross JA (2002). Gender Differences in First-Year Dental Students’ Motivation to Attend Dental School. J Dent Educ.

[29] Gallagher JE, Scambler S (2021). Reaching A Female Majority: A Silent Transition for Dentistry in the United Kingdom. Prim Dent J.

[30] Newton JT, Thorogood N, Gibbons DE (2000). A study of the career development of male and female dental practitioners. Br Dent J.

[31] Stewart FMJ, Drummond JR (2000). Women and the world of dentistry. Br Dent J.

[32] Gallagher JE, Eaton KA (2015). Health workforce governance and oral health: Diversity and challenges in Europe. Health Policy (New. York).

[33] Scarbecz M (2014). Students ’ Motivation to Attend Dental School.

[34] Al-Bitar ZB, Sonbol HN, Al-Omari IK (2008). Reasons for choosing dentistry as a career by Arab dental students. Eur J Dent Educ.

[35] American Dental Education Association, THE IMPORTANCE OF MANUAL DEXTERITY, (n.d.). https://www.adea.org/GoDental/Application_Prep/Preparing_for_Dental_School/The_Importance_of_Manual_Dexterity.aspx (Accessed 15 Feb 2022).

[36] Gillet D, Quinton A, Jeannel A (2002). Is there a link between writing ability, drawing aptitude and manual skills of dental students?. Eur J Dent Educ.

[37] Giuliani M, Lajolo C, Clemente L, Querqui A, Viotti R, Boari A, Miani CM (2007). Is manual dexterity essential in the selection of dental students?. Br Dent J.

[38] Bax NDS, Godfrey J (1997). Identifying core skills for the medical curriculum. Med Educ.

[39] Zhang W, Swangnetr M, Bloom P (2014). Advances in Physical Ergonomics and Human Factors: Part I.

[40] Imam S.Z (2020). Manual dexterity : an important tool for dentists.

[41] Jalink MB, Goris J, Heineman E, Pierie JPEN, Ten Cate Hoedemaker HO (2014). The effects of video games on laparoscopic simulator skills. Am J Surg.

[42] AnkayYilbas A, Canbay O, Akca B, Uzumcugil F, Melek A, Calis M, Vargel İ (2019). The effect of playing video games on fiberoptic intubation skills. Anaesth. Crit. Care Pain Med.

[43] Kennedy MM (2005). Close window to return to IVIS Managing Change : Understanding the Demographics of the Evolving Workforce. AAEP Proc.

[44] Rudolph CW, Rauvola RS, Costanza DP, Zacher H (2021). Generations and Generational Differences: Debunking Myths in Organizational Science and Practice and Paving New Paths Forward. J Bus Psychol.

[45] D.O.C.S. Career, A. Predict, O. An, A. From, L. Study, Analysis a, (1993) 368–370.

[46] Dentists : Occupational Outlook Handbook: : U.S. Bureau of Labor Statistics, (n.d.). https://www.bls.gov/ooh/healthcare/dentists.htm (Accessed 24 Feb 2022).

[47] 2022’s 100 Best Jobs in America | Best Jobs Rankings | US News Careers, (n.d.). https://money.usnews.com/careers/best-jobs/rankings/the-100-best-jobs (Accessed 24 Feb 2022).

[48] World Economic Outlook Database, April 2019, IMF.Org. (n.d.). https://www.imf.org/external/pubs/ft/weo/2019/01/weodata/weoselco.aspx?g=2200&sg=All+countries+%2F+Emerging+market+and+developing+economies (Accessed 12 Mar 2022).

[49] Devlin H, Giannini P (2005). Financing student education in the future. Br Dent J.

[50] Aggarwal A, Mehta S, Gupta D, Sheikh S, Pallagatti S, Singh R, Singla I (2012). Dental Students’ Motivations and Perceptions of Dental Professional Career in India. J Dent Educ.

[51] Milton CL (2012). Altruism. Nurs Sci Q.

[52] Wiesing U (2020). The Hippocratic Oath and the Declaration of Geneva: legitimisation attempts of professional conduct. Med Heal Care Philos.

[53] Khami MR, Murtomaa H, Jafarian M, Vehkalahti MM, Virtanen JI (2008). Study motives and career choices of Iranian dental students. Med Princ Pract.

[54] Chikte UME, students,  (1992). to the University of Sydney. Australia.

[55] Krueger RA, Casey MA (2000). Focus groups: A practical guide for applied researchers.

[56] Von Seggern M, Young NJ (2003). The focus group method in libraries: Issues relating to process and data analysis. Ref Serv Rev.

